# Identification of Potential JNK3 Inhibitors: A Combined Approach Using Molecular Docking and Deep Learning-Based Virtual Screening

**DOI:** 10.3390/ph16101459

**Published:** 2023-10-13

**Authors:** Chenpeng Yao, Zheyuan Shen, Liteng Shen, Kailibinuer Kadier, Jingyi Zhao, Yu Guo, Lei Xu, Ji Cao, Xiaowu Dong, Bo Yang

**Affiliations:** 1 Zhejiang Province Key Laboratory of Anti-Cancer Drug Research, Institute of Pharmacology and Toxicology, College of Pharmaceutical Sciences, Zhejiang University, Hangzhou 310058, China; chenpeng.yao@hotmail.com (C.Y.); 22119178@zju.edu.cn (K.K.); caoji88@zju.edu.cn (J.C.); 2 Innovation Institute for Artificial Intelligence in Medicine, Zhejiang University, Hangzhou 310058, China; 182800@zju.edu.cn (Z.S.); 22219164@zju.edu.cn (L.S.); 3 Hangzhou Institute of Innovative Medicine, College of Pharmaceutical Sciences, Zhejiang University, Hangzhou 310058, China; 22219170@zju.edu.cn (J.Z.); yu.guo@zju.edu.cn (Y.G.); 4 Institute of Bioinformatics and Medical Engineering, School of Electrical and Information Engineering, Jiangsu University of Technology, Changzhou 213001, China; leixu@jsut.edu.cn

**Keywords:** JNK3, hybrid virtual screening, binding pose metadynamics, molecular dynamics, bioactivity evaluation

## Abstract

JNK3, a member of the MAPK family, plays a pivotal role in mediating cellular responses to stress signals, with its activation implicated in a myriad of inflammatory conditions. While JNK3 holds promise as a therapeutic target for neurodegenerative disorders such as Huntington’s, Parkinson’s, and Alzheimer’s diseases, there remains a gap in the market for effective JNK3 inhibitors. Despite some pan-JNK inhibitors reaching clinical trials, no JNK-targeted therapies have achieved market approval. To bridge this gap, our study introduces a sophisticated virtual screening approach. We begin with an energy-based screening, subsequently integrating a variety of rescoring techniques. These encompass glide docking scores, MM/GBSA, and artificial scoring mechanisms such as DeepDock and advanced Graph Neural Networks. This virtual screening workflow is designed to evaluate and identify potential small-molecule inhibitors with high binding affinity. We have implemented a virtual screening workflow to identify potential candidate molecules. This process has resulted in the selection of ten molecules. Subsequently, these ten molecules have undergone biological activity evaluation to assess their potential efficacy. Impressively, molecule compound 6 surfaced as the most promising, exhibiting a potent kinase inhibitory activity marked by an IC_50_ of 130.1 nM and a notable reduction in TNF-α release within macrophages. This suggests that compound 6 could potentially serve as an effective inhibitor for the treatment of neuroinflammation and neurodegenerative diseases. The prospect of further medicinal modifications to optimize compound 6 presents a promising avenue for future research and development in this field. Utilizing binding pose metadynamics coupled with molecular dynamics simulations, we delved into the explicit binding mode of compound 6 to JNK3. Such insights pave the way for refined drug development strategies. Collectively, our results underscore the efficacy of the hybrid virtual screening workflow in the identification of robust JNK3 inhibitors, holding promise for innovative treatments against neuroinflammation and neurodegenerative disorders.

## 1. Introduction

JNK3, or c-Jun N-terminal kinase 3, is a member of the mitogen-activated protein kinase (MAPK) family, which plays a crucial role in regulating cellular responses to various stress signals, including inflammation. JNK1 and JNK2 are ubiquitously expressed, while JNK3 is predominantly expressed in the brain, and its activation has been implicated in numerous inflammatory conditions. The activation of JNK3 is carried out by two MAP kinases, MKK4 and MKK7. Upon activation, JNKs can interact with the pathway’s final effectors, phosphorylate transcription factors, such as the transcription factor activator protein-1 (AP1) family proteins, c-JUN, activating transcription factors (ATF), and (ETS Like-1 protein) Elk1 [1]. Based on knockout experiments and its specific tissue expression, JNK3 has emerged as a promising target for potential treatments of neuroinflammation and neurodegenerative disorders like Huntington’s disease [2,3,4], Parkinson’s disease [5], and Alzheimer’s disease [6,7,8,9,10,11,12,13].

Currently, pharmacologists have made many efforts in the field of JNK-targeted therapy, and some pan-JNK inhibitors have already been discovered. Two molecules are currently in clinical trials [14]. However, there are still no JNK inhibitors on the market. Of note, SP600125 is a typical pan-JNK inhibitor and is widely used as a positive control despite its poor selectivity [15]. As an ATP-competitive pan-JNK inhibitor, PGL5001 (also known as AS602801) is currently in phase Ⅱ clinical trials (NCT01630252) for the treatment of inflammatory endometriosis [16,17]. Tanzisertib, a second-generation ATP-competitive pan-JNK inhibitor, was discovered for the treatment of acute myeloid leukemia (AML) and is in phase Ⅰ clinical trials (NCT00126893) [18,19]. CC-930 was also discontinued in phase Ⅱ due to an increased risk of liver injury (NCT01203943) [20,21]. As far as we know, the development of a JNK3 inhibitor is difficult and there are no drugs on the market. Therefore, it is important to discover new JNK3 inhibitors with novel designs for the treatment of relevant indications.

In recent years, computational methods have been widely used to screen and design new drug candidates for various targets. Despite their growing popularity, existing virtual screening methods often suffer from limitations in accuracy [22]. It has been reported that the hit rate of virtual screening can be significantly improved by combining AI methods [23]. For instance, in a study focused on human topoisomerase I inhibitors, researchers combined machine learning models with virtual screening and molecular docking, successfully enhancing the hit rate of virtual screening and providing novel insights for the development of topoisomerase I inhibitors [24]. In another example, geometric deep learning-based algorithms were utilized to predict the binding affinity of small molecules to their protein targets, which significantly improved the performance of virtual screening campaigns and led to the identification of novel bioactive compounds [25]. Virtual screening typically operates in a hierarchical workflow, employing different methods in succession, each acting as a filter to discard unwanted compounds. This allows the advantages of various methods to be utilized while avoiding their limitations. However, research teams often only use techniques with which they are familiar. The results of virtual screening require experimental testing in the lab to confirm their biological activity. This can lead to false positive results. And many software programs are used for virtual screening, and because they use different algorithms, using the same input in different software can yield different results. Despite this, virtual screening remains a crucial tool in the drug discovery process. It significantly reduces the time and resources required for laboratory testing. This is because it allows for the rapid and efficient identification of potential drug candidates from large databases of compounds, thereby streamlining the initial stages of drug development.

**Figure 1:**
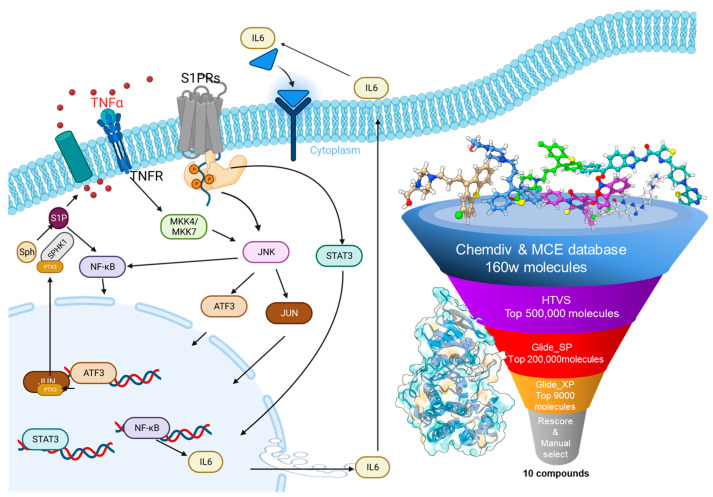
The signal transduction pathways and the hybrid virtual screening process of the JNK3 target.

In this study, we systematically applied a stepwise virtual screening methodology, first utilizing an energy-based screening approach, followed by a data-driven rescoring process. This intricate approach leveraged varying levels of glide docking accuracy, utilizing tools such as MM/GBSA, Deepdock, and an advanced Graph Neural Network to rescore our initial screening outcomes. Through this hybrid virtual screening workflow, we successfully pinpointed several candidates with promising JNK3 inhibitory potential. Notably, our best-performing molecule, boasting a kinase inhibition IC_50_ of 130.1 nM, underwent rigorous testing to assess its impact on the secretion of inflammation-associated cytokines IL-6 and TNFα in macrophages. Encouragingly, experimental findings indicated a significant reduction in TNF-α release, reinforcing the efficacy and dependability of our meticulously designed screening workflow in isolating potent JNK3 inhibitors (refer to Figure 1 for a visual representation) [26]. To deepen our understanding and provide a foundation for future drug design, we employed binding pose metadynamics and molecular dynamics simulations. This allowed us to elucidate the intricate binding dynamics between our top molecule and its target, setting the stage for optimized molecule development.

## 2. Results

### 2.1. Data Collection and “in-House” Database Construction

The datasets employed in this study consist of two parts: the JNK3 inhibitor activity dataset, containing 1072 molecules which were obtained from the ChEMBL database, is used for training, evaluation, and testing the performance of the rescoring module; the “in-house” database, assembled using the MCE-like compound library and Chemdiv compounds, which contains about 1,600,000 molecules, serves the screening task. The JNK3 inhibitor activity dataset has pIC_50_ as its label. Both datasets undergo preprocessing with RDKit to eliminate duplicate molecular structures, while Schrödinger’s LigPrep module generates the 3D structures of the molecules [27]. 

### 2.2. The Preparation of Protein and the Binding Pocket Determination

**Figure 2:**
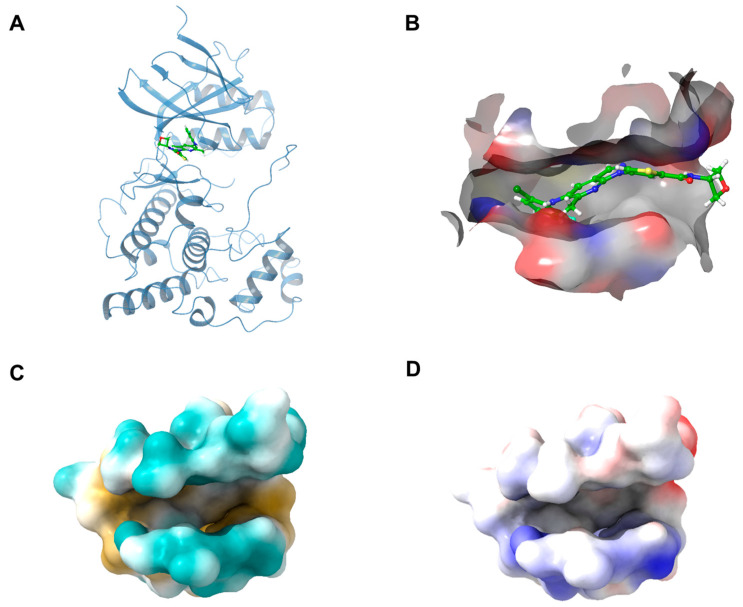
(**A**) The 3D binding mode of the JNK3 with its original ligand 4-[5-(2-chloro-6-fluoroanilino)-6-methyl-1H-pyrazolo[3,4-b] pyridin-1-yl]-N-(oxetan-3-yl) thiophene-2-carboxamide (PDB ID: 7S1N). (**B**) The binding conformation of the ligand in JNK3 complex. (**C**) The hydrophobicity of the binding pocket. (**D**) The electricity of the binding pocket.

Initially, the protein structure of JNK3 (PDB ID: 7S1N) [28] was obtained from RCSB (https://www.rcsb.org/) and well prepared with the Protein Preparation Wizard, Schrödinger 2021-suite. The binding pocket used for screening is determined by the location of the ligand in the JNK3 complex (Figure 2). Receptor grid surface features in the energy-based virtual screening and rescoring sessions were obtained from the Schrödinger suite of receptor grid generation and deep learning algorithm Masif calculations, respectively [29].

### 2.3. The Hybrid Virtual Screening Workflow

For our primary screening, the in-house database serving as our library undergoes a meticulous screening using the HTVS precision, all under the guidance of the OPLS_2005 force field [30]. Next, 500,000 molecules with docking scores smaller than −6.532 kcal/mol are selected for further accurate screening with SP precision under the OPLS4 force field. After this process, 200,000 molecules with scores ≤ −8.689 kcal/mol are retained. The final round of docking is performed with XP precision using the OPLS4 force field, resulting in 9000 molecules with scores ≤ −8.101 kcal/mol that are ultimately selected for the subsequent data-driven rescoring stage [31,32,33,34,35,36,37] (Table 1).

In the second part of the hybrid screening workflow, we initially docked and rescaled the characterized JNK3 pocket with the JNK3 inhibitor molecular dataset on ChEMBL using the Deepdock algorithm. Subsequently, the inhibitor molecular dataset was transformed into a graph, and the results of Deepdock’s scoring were normalized with molecular properties described by RDKit, such as TPSA, MW, and rotatable bonds, which were incorporated into the nodes as additional features. These node features were then fed into the GNN scoring model we developed, which consists of multiple GAT layers and a single MLP (Multilayer Perceptron) layer.

**Figure 3:**
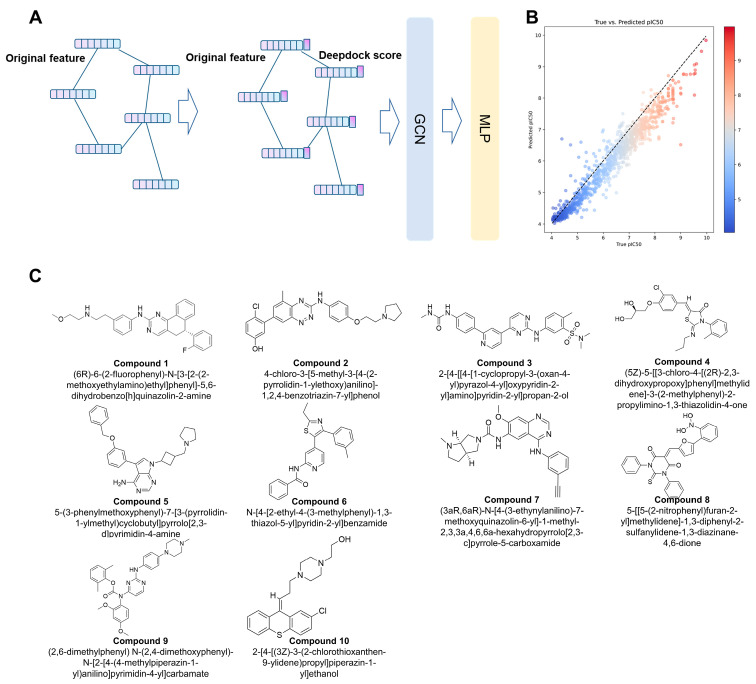
(**A**) represents the prediction by MLP after Deepdock scoring is added to the atomic feature vector and input to the GNN model for training. (**B**) represents the effect of the model on the dataset. (**C**) The structures and IUPAC names of the final ten molecules.

By utilizing the pIC_50_ data corresponding to each molecule as labels, the dataset was divided into training, validation, and test sets in a ratio of 8:1:1, allowing for the training of a regression task on the GNN model. The performance of the model was promising, with an R2 of 0.9617 on the training set, 0.7866 on the validation set, and 0.7228 on the test set, demonstrating its strong predictive capabilities (Figure 3).

To aid in decision-making, we also employed the energy-based rescoring method, MM/GBSA, which was conducted using the Schrödinger Suite’s Prime module. In the final analysis, we took into account the drug-like properties of the molecules, the binding conformations of the ligands to their respective targets, and the diverse scoring outcomes. Significantly, the selection methodology employed a meticulous assessment of both scoring metrics and molecular interactions to eliminate erroneous binding mode candidates. Additionally, leveraging the expertise of medicinal chemists, an estimation was made to identify molecules with superior binding affinities, a process that may have led to the exclusion of compounds not meeting drug-like criteria. Ultimately, ten molecules were chosen from the scoring results for subsequent biological experimental validation. (Table 1 and Table 2, Figure 3C).

### 2.4. Inhibitory Activity Assay

**Figure 4:**
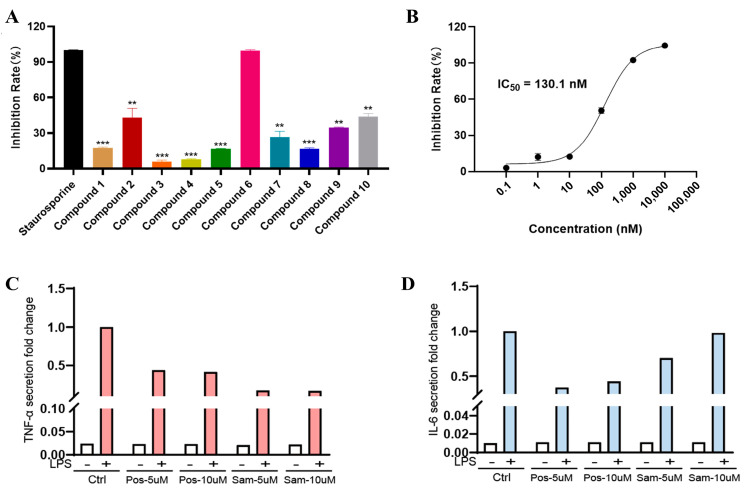
(**A**) The inhibition rate (%) assay of 10 selected compounds against JNK3 (Staurosporine is the reference compound); (**B**) the IC_50_ evaluation of compound 6; (**C**) TNF-α secretion study. Pos: JNK Inhibitor VIII, Sam: compound; (**D**) IL6 secretion study. ** represents a *p*-value less than 0.01; *** represents a *p*-value less than 0.001.

Based on the hybrid virtual screening result, the inhibitory activity of the 10 selected compounds was further assessed at a concentration of 25 μM. As depicted in Figure 4A, compound 6 demonstrated the highest inhibition rate in comparison to the other compounds. We proceeded to determine the IC_50_ value of compound 6 (Figure 4B), which exhibited potent inhibitory activity against JNK3 (IC_50_: 130.1 nM). This finding suggests that compound 6 possesses significant activity against JNK3.

### 2.5. Pro-Inflammatory Factor Release Inhibition Assay for Compound 6

Based on the results of the kinase activity, the secretion of TNF-α and IL-6 was further studied for compound 6. As shown in Figure 4C, compound 6 exhibited stronger inhibition of TNF-α secretion and slight inhibition of IL-6 secretion compared to the positive control (JNK inhibitor VIII). The combined experimental results suggest that compound 6 could be an ideal and popular compound for the design of novel JNK3 inhibitors with high biological activity.

### 2.6. Elucidating the Initial Binding Conformation of JNK3 and Compound 6 through Binding Pose Metadynamics

**Figure 5:**
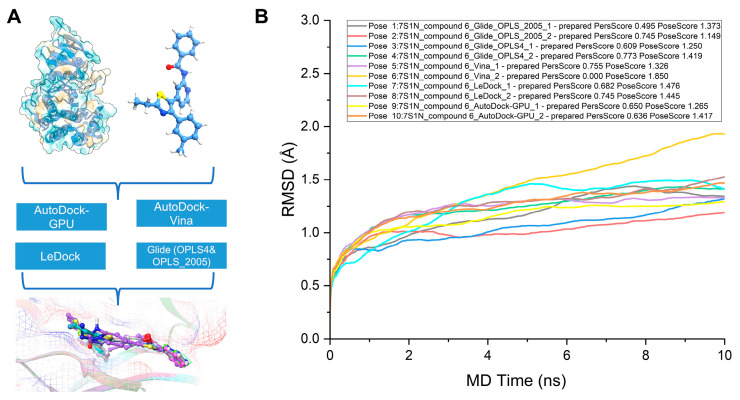
Schematics and results of metadynamics simulations. (**A**) Workflow of the metadynamics simulation. (**B**) The RMSD plot from diverse initial conformations of compound 6.

In this experiment, our objective was to identify the most stable initial conformation of JNK3 bound to compound 6 by employing a combination of various docking software and metadynamics simulations. We generated a diverse set of initial conformations using AutodockGPU, Autodock Vina, LeDock, and Glide_SP, incorporating both the OPLS2005 and OPLS4 force fields (Figure 5A). To further refine these conformations, we utilized Schrödinger’s binding pose metadynamics, conducting short metadynamics simulations in parallel for ten distinct groups of each complex. Our analysis revealed that the second conformation produced by Glide_SP, employing the OPLS2005 force field, demonstrated the greatest stability among all conformations. This determination considered its RMSD value, PersScore, and PoseScore, as shown in Figure 5B.

### 2.7. Structural Insight into the Precision Binding Mode between JNK3 and Compound 6 through Molecular Dynamics

**Figure 6:**
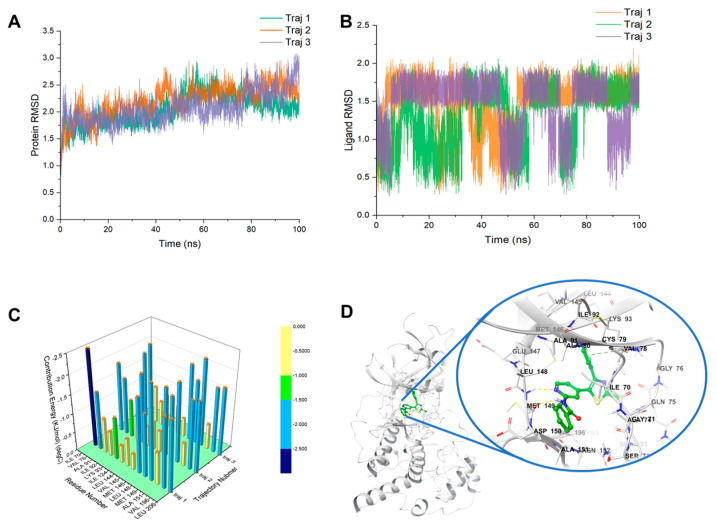
(**A**) RMSD value of JNK3; (**B**) RMSD value of compound 6; (**C**) the contribution of compound 6-JNK3 complex; (**D**) the 3D diagram of protein–ligand interaction.

We performed three parallel sets of 100 ns molecular dynamics simulations on the most stable docked conformation, based on the results of binding pose metadynamics, using the Amber dynamics module. As depicted in Figure 6A,B, the RMSD of both the protein and ligand reached a steady state during the final 20 ns. We analyzed the conformations from the last 1 ns of the three trajectories and employed MM/GBSA to compute the contribution of different amino acids to the binding energy under free energy decomposition. This analysis aimed to explore the explicit binding mode of compound 6 to JNK3 (Figure 6C). Our findings revealed that the N atoms of pyridine and amide can form two strong H-bond interactions with Met149. Additionally, the nitrogen atom of thiazolyl can generate an extra hydrogen bonding interaction with Lys93, facilitated by a water molecule. Simultaneously, the tolyl group of compound 6 can insert into specific hydrophobic pockets surrounded by Ala91, Ile92, Leu144, Val145, and Met146, resulting in robust hydrophobic interactions (Figure 6D). These specific interactions contribute to the high inhibitory activity of compound 6 against JNK3.

## 3. Discussion

The JNK3 pathway is a prominent druggable target that has received extensive research attention, with some inhibitors currently undergoing clinical investigation. Importantly, JNK3 has been established as a promising therapeutic target for addressing neurodegenerative disorders, signifying its substantial therapeutic potential. In line with contemporary trends in drug discovery, virtual screening coupled with artificial intelligence has emerged as a widely embraced method due to its cost-effectiveness and high efficiency. Accordingly, we have incorporated this innovative approach into our study.

In this study, we employed a multistage screening workflow aimed at identifying potential inhibitors. To assess the binding affinities of the compounds, we also implemented advanced scoring methodologies. This approach integrates energy-based screening methods, including the Glide docking tool, MM/GBSA, and data-driven rescoring techniques such as Deepdock and the advanced Graph Attention Networks mode. Our comprehensive approach, complemented by drug–target interaction analysis and an assessment of drug-like properties, led to the successful discovery of several potent JNK3 inhibitors. Among them, the standout inhibitor, compound 6 (N-(4-(2-ethyl-4-(m-tolyl)thiazol-5-yl)pyridin-2-yl)benzamide), displayed an impressive IC_50_ of 130.1 nM. Furthermore, it significantly curtailed the release of both IL-6 and TNFα, reinforcing the reliability and efficacy of our screening approach.

In a subsequent phase, we turned to binding pose metadynamics and molecular dynamics simulations to elucidate the intricate binding dynamics of compound 6 with JNK3. Our analyses spotlighted that the N atoms of pyridine and amide in the compound forged two potent H-bond interactions with Met149. Concurrently, the nitrogen atom of thiazolyl set up an additional hydrogen bond interaction with Lys93, a process facilitated by a water molecule. Notably, compound 6’s tolyl group adeptly nestled itself into specific hydrophobic pockets, defined by residues such as Ala91, Ile92, Leu144, Val145, and Met146, culminating in sturdy hydrophobic interactions. These nuanced interactions underscore the heightened inhibitory prowess of compound 6 against JNK3.

This study has successfully identified a novel JNK3 inhibitor with a distinctive molecular scaffold, thereby establishing a valuable foundation for the design and development of JAK3 inhibitors. However, as a lead compound, it holds the potential for further structural modification and selectivity enhancement. To fully validate their therapeutic potential, it is imperative to pursue further optimization and conduct rigorous in vivo and in vitro testing.

## 4. Materials and Methods

### 4.1. Protein Preparation

The protein structure (PDB ID: 7S1N) was obtained from RCSB protein database and given further preparation. The protein preparation steps were performed through the Protein Preparation Wizard module [38,39] in the Schrödinger Suite including the Import and Process, Review and Modify, and Refine steps. In the “Import and Process” step, problems in the structure were checked and fixed according to the default parameters, and the missing side chains and loops of the protein were filled in. In the “Review and Modify” step, we removed the water molecules and dealt with the incorrect information in the structure. Finally, we performed hydrogen bond optimization and energy minimization for the structure.

### 4.2. Database Preparation

The ligand database used for screening consists of two components, including the commercial compound library ChemDiv and MCE’s drug-like compound library. The LigPrep module, Schrödinger 2021-2 suit, was adopted to handle these molecules, including hydrogenation, salt removal, tautomer generation, and ionization states calculation at pH 7.0 ± 2.0, under the OPLS4 force field. Furthermore, under the computation condition of retaining specific chirality, each ligand can generate up to 32 stereoisomers.

### 4.3. Receptor Grid Generation

This step was to create [40] the corresponding file for further docking work based on the center coordinates of the protein pocket. In the Glide_SP step, the Schrödinger’s receptor grid generation module was invoked for this process, by taking the center coordinates of the original ligand as the center coordinates for the docking process and generating the corresponding grid file after removing the original ligand.

### 4.4. The Hybrid Virtual Screening Workflow

Our study’s hybrid virtual screening workflow is structured into two distinct phases: an initial energy-based screening followed by a data-driven rescoring. In the energy-based phase, we employ the Glide docking tool from the Schrödinger suite, which provides three precision levels: HTVS, SP, and XP. In the energy-based virtual screening phase, all docking was performed using the Glide_SP tool, with the maximum output conformations set to 5 for HTVS precision and 20 for both SP and XP. In the data-driven rescoring phase, the Deepdock scoring stage first utilized all ligand poses derived from the previous screening round. The binding energy between ligands and targets was evaluated using a pan-density hybrid network calculation and characterized into atomic nodes. The Graph Neural Network (GNN) model consists of multiple Graph Attention Network (GAT) layers and a single Multilayer Perceptron (MLP) layer, implemented using the DGL packages. Parameter optimization was performed using the Optuna tool.

### 4.5. IC_50_ Assay

Initially, the tested compound was dissolved in DMSO to make a 10 mM stock solution, and the stock solution was then diluted to a drug solution with 50× test concentrations for later use, wherein the test concentrations were reached through dilution at a 10-fold gradient and were 0.1 nM, 1 nM, 10 nM, 100 nM, 1000 nM, 10,000 nM, respectively. Firstly, 2× ATP and substrate solution and 2× kinase and metal solution was prepared using assay buffer (MgCl2 2 mM, MnCl2 1 mM, SEB 12.5 nM, DTT 0.5 mM). From each well of the 96-well plate, 25 μL of the drug solution was taken and then transferred to a 384-well plate which was provided with 2 duplicate wells. Then, 2.5 μL of 2× kinase and metal solution was mixed and incubated in a polystyrene-coated 384-assay plate for 10 min at 25 °C. Then, 2× XL665 and antibody solution was prepared with detection buffer. An amount of 5 μL of Kinase Detection Reagent was added to the well, and incubated for 60 min at 25 °C. The fluorescence signals of 620 nm (Cryptate) and 665 nm (XL665) were read by a microtiter-plate reader. At last, the IC_50_ value of JNK3 kinase was calculated with the following equations.

Y = Bottom + (Top-Bottom)/(1 + 10^((LogIC50-X) * hillslope))(1)

### 4.6. Pro-Inflammatory Factor Release Inhibition Assay in LPS-Induced RAW264.7

RAW264.7 cells were cultivated with DMEM supplemented with 10% FBS at 37 °C in a 5% CO_2_ incubator. Then, cells with a density of 2 × 105 cells/mL were seeded in 24-well plates and incubated for 12 h. Subsequently, the cells were treated with tested compound (5 µM and 10 µM) for 24 h and exposed to LPS (10 ng/mL) for another 24 h. After 24 h, the supernatant was collected, and the levels of IL-6 and TNF-α in the supernatant were measured using an ELISA kit. Then, cover each ELISA plate with 100 µL/well of capture antibody in Coating Buffer(1X), seal the plate and incubate overnight at 4 °C. The next day, remove the primary antibody and wash 3 times with >500 µL/well Wash Buffer (1X PBS, 0.05% Tween). Block wells with 200 µL of ELISA/ELISPOT Diluent (1X), incubate at room temperature for 1 h. Following incubation, discard the blocking solution and wash at least once with Wash Buffer. Then, configure the standard curve and add 100 µL/well of samples to the appropriate wells, incubate at room temperature for 2 h or overnight at 4 °C. After that, remove the samples and wash 3 times with Wash Buffer, and add 100 µL/well diluted Detection Antibody to all wells. Incubate the plate at room temperature for 1h. Next, remove the secondary antibody and wash the wells with Wash Buffer, followed by the addition of 100 µL/well of Streptavidin-HRP(1X), incubate the plate for 30 min at room temperature, and then wash the wells with double-distilled water; next, add 80 µL/well of 1X TMB solution, incubate at room temperature for 15 min, and then, stop the reaction by adding 1 M H_2_SO_4_. Finally, wipe the bottom of the plate and measure the absorbance values at 450 nm (reference wavelength: 570 nm) using a microplate reader. Calculate cytokine concentrations based on the absorbance values and compare the cytokine changes with the control group.

### 4.7. Binding Pose Metadynamics

The binding pose metadynamics (BPMD) is an automated, metadynamics-based method that ensures the ligand maintains its binding pose during simulation. Ten individual 10 ns simulations are performed, using the root-mean-square deviation (RMSD) of the ligand’s heavy atoms relative to their starting positions as the collective variable (CV). Before metadynamics runs, the system is solvated and gradually heated to 300 K, removing poor contacts and strain in the initial structure.

Ligand stability is assessed by two main scores: PoseScore, which represents the average RMSD from the starting pose, and PersistenceScore (PersScore), measuring the persistence of hydrogen bonds in the final 2 ns of the simulation. PersScore ranges from 0 to 1, where a higher value indicates preserved interactions between the initial ligand pose and the final 2 ns of the simulations.

### 4.8. Molecular Dynamics

The compound 6 with the highest score was utilized for further molecular dynamics simulations to investigate the interactions. All operations were performed under the AMBER force field: the ff19SB force field for the protein and GAFF for the ligand.

The complexes of JNK3 and compound 6 charge were neutralized with Na^+^ counterions. The system was then soaked in a truncated octahedral box of TIP3P water molecules extending to at least 15 Å from the protein atoms. The ff19SB force field was used to describe the protein, together with the general Amber force field (GAFF) [41] parameters for the ligand. The simulation was carried out using the Amber 19 Molecular Dynamics package [42]. The system, prepared as described above, was first subjected to 500 steps of steepest descent and 500 steps of conjugate gradient minimization. This was followed by an additional 2500 steps of steepest descent and 2500 steps of conjugate gradient minimization. The system was then heated to the target temperature of 300 K for a period of 20 ps under constant volume periodic boundary conditions (NVT). Subsequently, approximately 40 ns of constant pressure and temperature simulation (NPT) was carried out to equilibrate the system, which was followed by 30 ns of production simulation performed under the same conditions. An average pressure of 1 atm was maintained by using isotropic position scaling with a relaxation time of 2 ps. Temperature was controlled via Langevin dynamics. A cutoff of 10 Å was used for nonbonded interactions and long-range electrostatic interactions were treated by means of the Particle Mesh Ewald (PME) method. All bonds involving hydrogen were constrained by the SHAKE method and the time step for numerical integration was 1 fs. The simulation results were analyzed using the ptraj program in the Amber19 package.

## Figures and Tables

**Table 1:** Each stage score and the inhibition rate of the final selected 10 small molecules.

Entry	Docking Score (HTVS) (kcal/mol)	Docking Score (SP) (kcal/mol)	Docking Score (XP) (kcal/mol)	MM-GBSA dG Bind (kcal/mol)	Deepdockscore	pIC_50_ Predicted	Inhibition Rate (%)
Compound 1	−11.107	−11.896	−13.871	−86.10	−185.12	6.25	17.50 ± 0.55
Compound 2	−9.373	−10.870	−11.918	−80.93	−169.34	5.89	43.24 ± 5.36
Compound 3	−9.190	−8.696	−10.808	−85.84	−154.42	6.84	5.70 ± 1.02
Compound 4	−9.023	−10.094	−8.959	−76.28	−90.94	5.34	7.77 ± 0.43
Compound 5	−8.941	−9.200	−8.537	−83.07	−140.73	5.35	16.58 ± 0.34
Compound 6	−8.401	−11.535	−13.065	−82.05	−173.73	5.82	99.73 ± 0.44
Compound 7	−8.147	−10.439	−10.690	−78.60	−172.77	6.20	26.88 ± 3.29
Compound 8	−7.853	−10.406	−8.840	−80.03	−116.72	5.30	16.76 ± 0.56
Compound 9	−6.757	−11.754	−13.694	−87.01	−183.26	6.34	34.81 ± 0.23
Compound 10	−8.571	−9.496	−9.765	−75.76	−97.41	5.33	43.76 ± 1.92

**Table 2:** Physicochemical characteristics of investigated compounds.

Entry	Molecular Weight	XLogP3-AA	Hydrogen Bond Donor Count	Hydrogen Bond Acceptor Count	Rotatable Bond Count	Heavy Atom Count	Topological Polar Surface Area
Compound 1	468.6	5.3	2	6	9	35	59.1
Compound 2	476.0	5.3	2	7	7	34	83.4
Compound 3	435.5	2.1	2	7	7	32	94.3
Compound 4	461.0	4.6	2	6	8	31	108.0
Compound 5	453.6	4.60	1	5	7	34	69.2
Compound 6	399.5	5.6	1	4	5	29	83.1
Compound 7	442.5	3.1	2	6	5	33	82.6
Compound 8	495.5	5.6	0	6	4	36	132.0
Compound 9	568.7	5.9	1	9	9	42	92.3
Compound 10	401.0	4.3	1	4	5	27	52.0

## Data Availability

The data supporting the reported results in this study can be found in the following publicly available sources. ChEMBL database: The bioactivity data and compound information were obtained from the ChEMBL database, which is an open-source resource for curated bioactivity data of small molecules and their targets. The database can be accessed at https://www.ebi.ac.uk/chembl/ (accessed on 13 April 2023). ChemDiv compound library: The ChemDiv compound library was used for the virtual screening of potential drug candidates. The library is a commercially available collection of diverse small molecules and can be accessed through the TargetMol company (https://www.targetmol.com/) (accessed on 13 April 2023). MCE (MedChemExpress) compound library: The MCE compound library, another commercially available collection of diverse small molecules, was also utilized in this study. The library can be accessed at https://www.medchemexpress.com/ (accessed on 13 April 2023).
